# Adherence to Dietary Recommendations in Organized Living Beneficiaries with Severe Mental Disorders and Their Caregivers

**DOI:** 10.3390/nu16010143

**Published:** 2023-12-31

**Authors:** Lidija Šoher, Milica Cvijetić Stokanović, Sara Prša, Maja Miškulin, Daniela Kenjerić

**Affiliations:** 1 Department of Food and Nutrition Research, Faculty of Food Technology Osijek, Josip Juraj Strossmayer University of Osijek, Franje Kuhača 18, 31000 Osijek, Croatia; lidija.soher@ptfos.hr (L.Š.); mcvijetic@ptfos.hr (M.C.S.); 2 Center for Providing Community Services Osijek „ME just like YOU“, Martina Divalta 2, 31000 Osijek, Croatia; sara.prsa10@gmail.com; 3 Faculty of Medicine Osijek, Josip Juraj Strossmayer University of Osijek, 31000 Osijek, Croatia; maja.miskulin@mefos.hr

**Keywords:** dietary intake, energy intake, dietary reference values, severe mental disorders, caregivers, organized living

## Abstract

People with severe mental disorders often require special care. Other than institutionalized care, some organizations provide housing options and special care in the form of organized living. Few studies provide a detailed description of nutrient intake in this type of care. The aim of this prospective study was to assess nutritional status and adherence to dietary recommendations in both people with mental disorders (beneficiaries) and their caregivers. Across three levels of care, 46 beneficiaries and 19 caregivers participated in the study. The mean intakes of energy (kcal/day) and macro- and micronutrients (g/day) were estimated from a 3-day dietary record and compared with dietary reference values (DRVs) set by the European Food Safety Authority (EFSA). The majority of participants did not meet energy intake recommendations (kcal/day). The contribution of total fat to energy intake (% E) was higher than recommended in both beneficiaries and caregivers with 42.1 ± 4.4% and 38.5 ± 6.5%, respectively. A total of 45.2% of beneficiaries and 61.1% of caregivers had their carbohydrate intake within the recommended range. Fiber intake was well below the recommendations (25 g/day) in all groups. Across all levels of care, lower micronutrient intake for pantothenic acid, biotin, folate, potassium, magnesium, copper, selenium, and iodine was observed. Based on the current results, the development and implementation of nutritional guidelines may be targeted to specific populations and nutrient intakes.

## 1. Introduction

The high prevalence of mental disorders, common onset in young adulthood, long-lasting nature, impact on life quality, and substantial contribution to healthcare burden make mental disorders a public health challenge worldwide. Severe mental disorders include bipolar disorder, major depressive disorder, schizophrenia, and schizoaffective disorder [1]. According to the data of the Croatian Institute of Public Health, currently, mental disorders account for approximately 4-5% of primary health care morbidity and 6% of total hospital morbidity in Croatia. By the number of hospitalizations, the most frequent subgroup of mental disorders in 2020 was the subgroup of schizophrenia, schizotypal, delusional, and other non-mood psychotic disorders [2]. The etiology of schizophrenic disorders is not fully understood and involves multiple factors, such as genetic, biological, and social factors. Treatment usually encompasses a combination of pharmacological and psychosocial interventions, depending on the disorder itself. Pharmacological therapy often implies the use of antipsychotics, antidepressants, sedatives, and anxiolytic drugs [3,4]. The use of antipsychotics coincides with metabolic side effects, such as dyslipidemia, hypertension, hyperglycemia, an increase in body weight and waist circumference, and a high percentage of adipose tissue [5,6,7]. The development of comorbidities such as obesity, cardiovascular diseases, and type 2 diabetes results in life expectancy shortening in this population by 8 to 20 years [3]. One of the proposed reasons for these side effects is the antipsychotics’ influence on dietary habits, the reduction in satiety, increased appetite, and increased cravings for sweet food and drinks [8]. 

Obesity is a serious problem in people with schizophrenic disorders. The prevalence of obesity in this population is 41–50% [5]. In addition to an elevated body mass index (BMI), studies of body composition have shown an increased proportion of fat tissue and a decreased proportion of muscle mass [7,9]. Several factors that may contribute to this are indicated in the literature: socioeconomic factors [10], use of tobacco products [11,12], poorer dietary habits [13], low physical activity [14], and metabolic disorders caused by antipsychotic therapy [7,15]. The poorer dietary habits are manifested through a high intake of fat, saturated fatty acids, sugar, and alcohol on one side and a reduced intake of fiber, fruits, vegetables, and fish on the other [11,12,13]. The average daily energy intake of individuals with schizophrenic disorder does not exceed that of the general population. Therefore, obesity can be the result of several causes, including an unhealthy lifestyle, high use of tobacco products, and antipsychotic therapy [13]. Moreover, people from the immediate vicinity (caregivers) often show similar eating habits [16]. A healthy diet is associated with mental well-being and a reduced risk of cognitive decline. Maintaining a healthy and balanced diet can play a vital role in preventing previously mentioned comorbidities as well as cognitive decline in people with severe mental disorders [3,8,13].

Apart from institutionalized care, which excludes them from society, people with severe mental disorders can receive medical help and social support through daily care provided by centers for community services in the form of community living, or living in organized housing. Unfortunately, very few studies provide detailed information on energy and nutrient intake in people with severe mental disorders living in this type of housing, as well as their caregivers. Therefore, the aim of this prospective study was to assess nutritional status and adherence to dietary recommendations in both people with severe mental disorders (beneficiaries) and their caregivers. The European Food Safety Authority (EFSA) established dietary recommendations for the EU through Dietary Reference Values (DRVs) [17]. 

## 2. Materials and Methods

### 2.1. Study Design and Population

This study was performed at the Department of Food and Nutrition Research at the Faculty of Food Technology Osijek and Center for Providing Community Services Osijek „ME just like YOU“, in October 2021. The center is a social care institution whose main activity is the integration of people with mental challenges into the community. Caregivers are responsible for the daily care of beneficiaries and participate in daily activities such as buying groceries and preparing and serving food in organized housing units. Depending on their diagnosis, beneficiaries are provided with 4 levels of care—occasional (2–3 times a week), daily short-term care (2.5 h/day), intensive (16 h/day), and comprehensive care (24 h/day)—and accommodated in about 30 housing units in the city of Osijek. In addition to the beneficiaries, caregivers were included in the study. 

Before the study began, both beneficiaries and caregivers were informed in detail about the course and purpose of the study. Depending on the level of guardianship, consent forms were signed. The study concept and methodology were approved by the Ethics Committee for Human Research of the Faculty of Food Technology Osijek (Class: 033-08/21-01/01; RegNo: 2158-82-01-21-58).

Participants included in the study were adults of both sexes, beneficiaries of the Center and organized housing or participating in the provision of services at the Center (caregivers), for whom written consent to participate in the research can be obtained. On the other end, participants were excluded from the study if they did not give or were unable to give, due to the severity of the disorder or cognitive and other impairments, their written consent and were unable to fulfill or follow the instructions related to the implementation of the study. The study protocol included the administration of questionnaires, anthropometric measurements, and dietary assessment through a dietary record. 

### 2.2. Administration of the Questionnaires

Using the face-to-face interview method, questionnaires created specifically for this study were administered. All questionnaires were created in cooperation with the Center’s psychologists and adapted to the population for which they were intended. Data on sociodemographic characteristics, lifestyle habits, and physical activity were collected. For physical activity level, beneficiaries were asked to self-assess their activity level with a 1–5 scale, and for estimation within the caregiver group, a short-form version of the International Physical Activity Level Questionnaire (IPAQ) was used [18]. The questionnaires were administered by a trained interviewer and, depending on the beneficiary’s condition, administered in written or verbal form, without changing the question’s aim or purpose. 

### 2.3. Anthropometric and Body Composition Measurements

Body mass index (BMI) and bioelectric impedance were used to determine nutritional status. Body weight (kg) and body composition were measured with an Omron BF500 analyzer (Omron Healthcare Co., Ltd., Kyoto, Japan). Body height (cm) was measured with a portable stadiometer seca 213 (Seca, Hamburg, Germany). The BMI was calculated as a ratio of body weight in kg to height in squared meters (kg/m^2^) and categorized according to World Health Organization (WHO) definitions as: underweight (<18.5 kg/m^2^), normal weight (18.5–24.9 kg/m^2^) and overweight (≥25.0 kg/m^2^) ), which was further categorized as preobese (25.0–29.9 kg/m^2^), obese class I (30.0–34.9 kg/m^2^), obese class II (35.0–39.9 kg/m^2^) and obese class III (≥40.0 kg/m^2^) [19]. Body composition was measured when possible due to pronounced tremors or other present disturbances. The percentage of body fat was interpreted according to the levels given by the manufacturer [20]. Body composition information was obtained for 39 beneficiaries.

### 2.4. Dietary Assessment

The data on dietary intake were collected using a dietary record. The dietary record was kept for three days including two weekdays and one weekend day. Not to further burden the participants, a period of three days was chosen. Participants, both beneficiaries and caregivers, were given clear and detailed instructions on keeping a 3-day dietary record (3-dDR). The days for which they kept a dietary record did not have to be consecutive. Along with face-to-face instructions given by a trained nutritionist, a premade form for keeping a record was given to all participants. To estimate portion size, standard household measurements (e.g., tablespoon, teaspoon, cup, glass, plate), food portions (e.g., a slice of bread), or packaging size were used. Besides household measures, standard food portions and picture books including a selection of country-specific dishes in different portion sizes were used. If a specific recipe or dish was consumed, participants were asked to describe in detail which ingredients were used, and how the dish was prepared. Dietary supplementation was also captured within the 3-dDR, encompassing the information on supplement type, dose, and manufacturer. To address any potential missing or overlooked entries, 3-dDRs were reviewed and crosschecked the day after recording with a caregiver. 

Energy intake and intake of 30 selected macro- and micronutrients were derived from 3-dDR. Data collected through dietary records were processed with the online software Program Prehrane 6.41.0 (IG PROG, Rijeka, Croatia) per subject. The mean daily intakes were calculated by dividing the total intake by the number of days in which the dietary record was kept. All dietary record procedures were conducted by a trained nutritionist and food technologist fully acquainted with the software and dietary record assessment. Participants’ mean energy and nutrient intake were compared with the DRVs proposed by the European Food Safety Authority (EFSA) [17]. When available to assess participants’ adherence to dietary recommendations, adequate intake (AI) or average requirement (AR) was used.

### 2.5. Statistics

The sociodemographic and anthropometric characteristics of participants by subgroup were presented as frequency and percentage or mean and standard deviation (SD) for the categorical and continuous variables, respectively. Dietary intake was presented as mean and SD followed by percentage of contribution to dietary recommendations. Data analyses and descriptive statistics were performed using the statistical software package Statistica version 14.0.1.25 (1984–2020 TIBCO Software Inc., Hamburg, Germany) and Microsoft Excel 2016 (version 16.0.5413.1000, 2016 Microsoft Corporation, Redmont, WA, USA). 

## 3. Results

### 3.1. Sociodemographic and Anthropometric Characteristics of Participants

In total, 65 participants completed questionnaires, 46 beneficiaries (57.0% female; 43% male) and 19 caregivers (all female). The sociodemographic and anthropometric characteristics of participants are presented in Table 1. Data on beneficiaries are stratified according to the three levels of care included in the study. Only 19.6% of beneficiaries had short-term care, and 54.3% of them had intensive daily care. The mean age was 46.4 ± 11.3 and 50.1 ± 7.7 years for beneficiaries and caregivers, respectively. Among all beneficiaries, ~70% smoked, with 62.5% of those in intensive care. Among caregivers, 63.2% of them were smokers. Most beneficiaries had medium-level education and were single or never married, whereas caregivers had medium-level education, but over half of them were married. In regard to mental disorder diagnosis, 80.0% of beneficiaries were diagnosed with schizophrenia. Several beneficiaries were diagnosed with moderate mental retardation, bipolar affective, or depressive disorder. A total of 37% of beneficiaries had been diagnosed with additional diseases. Among other diagnoses, the most often were hypertension and diabetes mellitus. When asked to estimate their physical activity, the most frequently selected answer on a 1-5 scale for beneficiaries was 3. For caregivers, IPAQ results showed that 52.6% of them had a high level of physical activity.

Using BMI as a criterion, underweight values were not recorded, neither for beneficiaries nor caregivers. A total of 80.3% of beneficiaries and 61.9% of caregivers were categorized as preobese or obese, 6.5% of beneficiaries were categorized as obese class III, and no class III obesity was observed in caregivers. In terms of level of care, all beneficiaries in comprehensive care had a BMI of 25.0 kg/m^2^ or above. The mean percentage of body fat was similar in both beneficiaries and caregivers with the mean being 38.6 ± 12.1% and 38.4 ± 6.6%, respectively. Both values are categorized as high or very high levels of body fat, depending on age and gender. The highest mean body fat was measured in short-term care. The mean visceral fat level is normal in caregivers but categorized as high in beneficiaries.

### 3.2. Energy and Macronutrient Intake and Adherence to Dietary Recommendation

Mean daily energy and macronutrient intake along with a proportion of participants who met the recommendation are presented in Table 2. Data on beneficiaries are presented in total and according to each level of care included in the study. Dietary reference values are also given for comparison. Out of the participants included in the study four beneficiaries and one caregiver’s data on the 3-dDR were missing. The mean energy intake was 1921.0 ± 391.5 kcal/day for beneficiaries and 1673.4 ± 263.7 kcal/day for caregivers. Regarding given recommendations, which vary according to age and gender, most participants did not reach the recommended energy intake. For beneficiaries, only 23.1% of female and 25.0% of male participants had an average energy intake equal to or above the set recommendations. In comprehensive care, 33% of male and female participants met the recommendations, the highest percentage of met recommendations in terms of care. The results showed that only 11.1% of caregivers met their energy requirements.

Recommendations for protein intake are given according to body mass. The mean protein intake was 70.9 ± 15.6 g/day and 62.3 ± 16.8 g/day, for beneficiaries and caregivers, respectively. Most participants (76.2% beneficiaries; 94.4% caregivers) met their protein requirements. In short-term care, a lower mean protein intake of 56.0 ± 7.5 g/day was recorded. In general, there was an excess of fat consumption, with over 30% accounted by saturated fat across all observed groups. The mean contribution of fat to daily energy intake of 42.1 ± 4.4% for beneficiaries and 38.5 ± 6.5% for caregivers exceeded the recommendations. On the other hand, the mean contribution of carbohydrates to energy intake is on the lower end of recommendations, ranging from 43.25 ± 4.7% in comprehensive care to 45.4 ± 3.7% in short-term care and 47.8 ± 6.8% for caregivers. Finally, 45.2% of beneficiaries and 61.1% of caregivers had their carbohydrate intake in the recommended range. Across all observed groups, dietary fiber intake was below recommendations (25 g/day). 

### 3.3. Micronutrient Intake and Adherence to Dietary Recommendations

Vitamin and mineral intake for both beneficiaries and caregivers is given in Table 3. Results are presented as mean daily intake and percentage of dietary recommendations. 

Among vitamins, mean daily intakes above recommendations, for beneficiaries were observed for vitamins B, thiamine, riboflavin, niacin, pyridoxine, and cobalamin. When stratified according to the level of care, the mean intake of vitamin E (11.3 ± 4.2 mg/day) and vitamin C (95.7 ± 72.1 mg/day) for female participants in intensive care met the recommendations. Additionally, the mean intake was above the recommendations for vitamin K in short-term and comprehensive care. Caregivers met the recommended intake of seven out of thirteen vitamins; mean daily intakes were below the recommendations for vitamin E, vitamin D, pantothenic acid, folate, and cobalamin. 

For minerals, intake below recommendations was observed in potassium, magnesium, copper, manganese, selenium, and iodine among all participants. An average daily sodium intake above recommendations (2 g/day) was observed in both beneficiaries and caregivers. Across levels of care, sodium intake ranged from 2.8 ± 0.6 g/day in short-term care to 4.3 ± 1.0 g/day in comprehensive care.

## 4. Discussion

The current study focused on people with severe mental disorders, primarily schizophrenia, residing in organized housing and their caregivers. The main finding of the present study is an overview of nutrient intakes and adherence to dietary recommendations of people with severe mental disorders and their caregivers. Additionally, a high BMI, a high prevalence of smokers, and sociodemographic characteristics that indicate lower socioeconomic status were observed. Results were compared with existing schizophrenia research, given that the majority of the beneficiaries had been diagnosed with schizophrenia. These results, to the best of our knowledge, are the first of this kind in Croatia.

Numerous studies showed a higher prevalence of smoking among schizophrenia patients [9,10,11,12,13]. In Jakobsen et al.’s [13] study, more than half of the participants (52.1%) reported daily smoking, whereas in Heald et al. [11], 64.9% were smoking. In the current study, the percentage of smokers was even higher, particularly in intensive care. People with schizophrenia have more than five times the odds of currently smoking compared to the general population [21]. In the present study, caregivers smoked in similar shares as beneficiaries. Results reported by Marthoenis et al. [9] showed that 36.2% of patients and 63.8% of the control group were married. Marriage rates among beneficiaries (4.4 %) were substantially lower than reported in previous research, unlike rates among caregivers (68.4%). The results of the current study are in accordance with the percentage of married patients in community-based Japanese patients with schizophrenia [22]. Lower education levels may lead to lower socioeconomic status, which may play a role in unhealthy dietary habits [12]. In the current study, half of the caregivers in our study and 63% of beneficiaries had a medium education level. Education level among patients with schizophrenia is usually lower [9,12]. Factors like smoking, a lower education level, physical inactivity, and a sedentary lifestyle are some of the most common risk factors for chronic non-communicable diseases, in both the general population and people with severe mental disorders [23,24]. In the current study, beneficiaries are encouraged by the caregivers to participate in daily activities, such as housework, grocery shopping, cooking, and taking longer walks a few times a week. This may explain the results showing that the most frequent level of self-perceived activity was moderately active. 

Results for underweight participants with schizophrenia varied in previous research [7,9,25,26,27]. With only 2% of the total sample categorized as underweight, the results of Costa et al. [12] are closer to the presented ones. The mean BMI of 31.0 ± 6.2 kg/m^2^ obtained in the current study for beneficiaries is in accordance with the mean BMI presented by Jacobsen et al. [13]. The majority of beneficiaries in the current study were overweight or obese, a predictable outcome, given the established prevalence of overweight and obesity in people with schizophrenia available in the literature [26,27]. When compared to caregivers (the control group), people with schizophrenia had higher BMIs [27,28]. The highest BMI and body fat percentage were measured among beneficiaries in short-term care. Patients with schizophrenia are known to have less healthy dietary habits and lower levels of physical activity due to lower socioeconomic status and suboptimal education levels or living conditions [11,12,13,29]. Drug-induced weight gain is also a contributing factor [12,30]. While previous research indicates a higher body fat percentage in schizophrenia patients compared to healthy individuals [30], our results showed similar body fat percentages in both beneficiaries and caregivers (38.6 ± 12.1%; 38.4 ± 6.6%). Similarly, Marthoenis et al. [9] found no difference between body fat percentage results between patients and healthy controls. In contrast, the results of this study did not show lower muscle mass in beneficiaries as suggested by previous studies, but it did reveal higher visceral fat levels compared to caregivers [9,28,30]. 

Considering daily energy intake, the results of the current study revealed an inadequate energy intake: 23.8% of beneficiaries met their specific energy requirements as opposed to 11.1% of caregivers. Among beneficiaries in short-term care, no one met their energy requirements. Energy intake under recommendations was previously reported [12,13,29,31]. In the general population, under-reporting in dietary intake assessment is a common and widely observed problem. Gender, older age, weight status, and smoking are some determinants associated with under-reporting in the general population. There is a lack of evidence in schizophrenia patients, but given cognitive and motivational challenges, under-reporting has to be taken into consideration. Lower energy intake may additionally emphasize the problem of increased body weight in this population. Multiple factors contribute to the increase in body mass, including antipsychotic therapy [12,13]. 

Previous studies on macronutrient contribution to total energy intake reported varied results [13,22,29,31]. The results of the current study showed an imbalanced distribution of macronutrients, in both beneficiaries and caregivers, in favor of fat. Jakobsen et al. [13] presented results that were under recommendations but exceeded them in saturated fat, sugar, and alcohol intake. In accordance, the current study demonstrated higher than recommended saturated fat intake in both beneficiaries and caregivers. Constant high fat intake, which favors saturated fat, can contribute to the development of higher levels of adipose tissue, elevated blood cholesterol, hyperlipidemia, and other cardiovascular conditions associated with schizophrenic disorders [5]. Polyunsaturated fats are known for their positive effects on mental health, cognitive performance, mood, stress, and neuroinflammation. A diet poor in polyunsaturated fats, often linked to the lower socioeconomic status of people with mental disorders, may present a challenge for dietary interventions [32]. Protein intake usually shows recommended values or above [12,13]. More than half of beneficiaries and almost all caregivers in the current study met their protein requirement; lower mean intake was recorded amongst beneficiaries in short-term care. Different results are shown for carbohydrate contribution. Ito et al. [22] presented higher carbohydrate contribution to energy intake in patients with schizophrenia, whereas in our study, despite mean carbohydrate intake falling within the recommendations, only half of the participants met their requirements. Dietary fiber, essential for digestion, may help prevent cardiovascular diseases [33] and improve mental health [34]. Fiber intake in both populations, with schizophrenic disorders and general populations, is lower than recommended [12,13,31,35]. 

Deficiencies of various nutrients, most often vitamins, are linked to impaired cognition. B vitamins like cobalamin, thiamine, riboflavin, pyridoxine, folate, and niacin have been associated with a range of mental health conditions, from depression and poor memory to mania and psychosis [29,35]. The results of the present study showed that apart from folates, both beneficiaries’ and caregivers’ mean daily intake met the recommendations. Men in short-term care had mean pyridoxine and riboflavin intake below the recommendations. Cobalamin, pyridoxine, and folate play an important role in neurotransmitter synthesis, the levels of neurotoxic homocysteine, and oxidative stress levels [35]. In terms of oxidative stress levels, the intake of vitamins C, E, and A may be considered important. In the present study, caregivers had mean vitamin C intake in line with the recommendations, possibly due to supplement consumption. A lower intake of vitamin C, as well as vitamin C deficiency, has been noticed in psychiatric patients [36]. In the present study, apart from vitamin K, mean daily intakes of fat-soluble vitamins are mostly deficient. Vitamin D intake is below recommendations in both beneficiaries and caregivers, which is in accordance with current reports. According to some authors, vitamin D deficiency may be a silent pandemic, affecting roughly 30–50% of the general population [37,38]. In regards to psychosis and schizophrenia, a lower intake of vitamin D in infantry may oppose a greater risk of developing these diseases in adulthood. In addition, a high intake of vitamin D may lower the rate of psychotic-like symptoms in people with severe mental disorders [37,38]. Stefańska et al. [39] detected a deficiency of vitamin D, E, C, folates, potassium, calcium, and magnesium in men and iron and iodine in women with schizophrenia. Apart from calcium and iron, the results obtained in the present study in the group of beneficiaries are similar. Magnesium [40], zinc [3,41], and other essential trace elements [42] were extensively studied regarding mental disorders. Even though results are not unanimous, the supplementation of magnesium in case of inadequate intake may be beneficial in people with mental disorders [40]. Trace minerals may influence mental disorders through various mechanisms. Further research on the safety and efficacy of micronutrient supplementation is needed [42]. A balanced diet, containing appropriate amounts of all vitamins and minerals, may still be the way to go when addressing intake below recommended values. Sodium and phosphorus intake in some cases were double the recommended value. High salt intake has already been recorded, in both people with schizophrenia [31] and the general population in Croatia [43]. It is important to take into account that hypertension is one of the most common accompanying diagnoses among beneficiaries in this study.

Previous studies have extensively addressed several limitations in evaluating and assessing dietary intake in people with severe mental disorders [12,13,26,29]. Selecting appropriate nutrition assessment tools, the reliability of collected data, participant recruitment, and motivation proved to be a challenge. By including caregivers, our research gained a new perspective and advantage. Caregivers participated in daily activities in housing units, reminded beneficiaries to record their food intake, and served as a surrogate source in case of any missing data. The inclusion of food supplements in nutrition assessment provides a more comprehensive overview of dietary intake. Besides enriching limited literature data on dietary intake in this population, the significance of the present study is additionally elevated due to the specific type of care—organized housing in comparison to hospitalization. Finally, the results presented in this study further highlight the vulnerability of this population. Despite the research’s limiting design, a comprehensive nutrition assessment showed that nutritional education is needed for both beneficiaries and caregivers. The development of multidisciplinary programs aimed at improving nutrition knowledge and the design of targeted nutritional interventions could lead to an improvement in the daily care and quality of life of people with severe mental disorders. 

## 5. Conclusions

Inadequate dietary intake remains a significant problem in people with severe mental disorders. This study showed that the diet of people with severe mental disorders (beneficiaries) and people in charge of their daily care (caregivers), for the most part, was not in line with dietary recommendations set by the EFSA. Therefore, future research focused on specific diagnoses and other factors that influence nutrition in this type of care and the development of dietary guidelines, along with adequate nutritional counseling, are needed in providing care for people with severe mental disorders, for both beneficiaries and caregivers. 

## Figures and Tables

**Table 1:** Participants’ sociodemographic and anthropometric characteristics, with beneficiaries stratified according to level of care.

	Beneficiaries (*N* = 46)	Short-Term Care (*n* = 9)	Intensive Care (*n* = 25)	Comprehensive Care (*n* = 12)	Caregivers (*n* = 19)
Gender, *n* (%)	♀ 26 (57.0); ♂ 20 (43 %)	5 (55.6)	15 (60.0)	6 (50.0)	♀ 19 (100)
Age (years) ^a^	46.4 ± 11.3	47.0 ± 9.1	47.4 ± 11.2	43.9 ± 13.4	50.1 ± 7.7
Smoking, *n* (%)	32 (69.6)	5 (55.6)	20 (80.0)	7 (58.3)	12 (63.2)
Education ^b^, *n* (%)					
Low education	14 (30.5)	2 (22.2)	6 (24.0)	6 (50.0)	2 (10.5)
Medium education	29 (63.0)	7 (77.8)	16 (64.0)	6 (50.0)	17 (89.5)
High education	3 (6.5)	-	3 (12.0)	-	-
Marital status, *n* (%)					
Single/Never married	33 (71.7)	8 (88.9)	16 (64.0)	9 (75.0)	-
Married	2 (4.4)	-	-	2 (16.7)	13 (68.4)
Divorced	7 (15.2)	1 (11.1)	5 (20.0)	1 (8.3)	4 (21.5)
In a relationship	3 (6.5)	-	3 (12.0)	-	1 (5.3)
Widowed	1 (2.2)	-	1 (4.0)	-	1 (5.3)
Diagnosis, *n* (%)					
Schizophrenia	37 (80.4)	7 (77.8)	21 (84.0)	9 (75.0)	
Depressive disorder	1 (2.2)	-	1 (4.0)	-	
Moderate mental retardation	5 (10.9)	1 (11.1)	2 (8.0)	2 (16.7)	
Psychosis	1 (2.2)	1 (11.1)	-		
Bipolar affective disorder	2 (4.3)	-	1 (4.0)	1 (8.3)	
Other diagnosis, *n* (%)	17 (37.0)	5 (55.6)	7 (28.0)	5 (41.7)	4 (21.1)
Supplements, *n* (%)	10 (21.7)	2 (22.2)	8 (32.0)	-	9 (47.4)
Physical activity level ^c^	3 (17)	4 (5)	3 (10)	3 (4)	1 (10)
Anthropometric characteristics					
Height, cm	168.7 ± 11.7	167.2 ± 12.2	169.9 ± 11.4	167.3 ± 12.7	164.6 ± 5.9
Weight, kg	87.9 ± 18.7	89.0 ± 18.5	85.1 ± 16.1	92.9 ± 24.0	73.6 ± 12.1
BMI (kg/m^2^)	31.0 ± 6.2	32.3 ± 8.2	29.6 ± 5.9	32.8 ± 4.7	27.2 ± 4.6
Underweight, n (%)	-	-	-	-	-
Normal weight, n (%)	9 (19.6)	3 (33.3)	6 (24.0)	-	6 (28.6)
Overweight/preobese, n (%)	14 (30.4)	-	9 (36.0)	5 (41.7)	11 (52.4)
Obese class I, n (%)	10 (21.7)	2 (22.2)	5 (20.0)	3 (25.0)	-
Obese class II, n (%)	10 (21.7)	3 (33.3)	3 (12.0)	4 (33.3)	2 (9.5)
Obese class III, n (%)	3 (6.5)	1 (11.1)	2 (8.0)	-	-
Body composition ^a^	(*n* = 39)	(*n* = 9)	(*n* = 22)	(*n* = 8)	(*n* = 19)
Body fat (%)	38.6 ± 12.1	41.2 ± 15.2	37.6 ±11.6	38.3 ± 10.8	38.4 ± 6.6
Muscle mass (%)	27.4 ± 6.2	26.1 ± 7.3	27.7 ± 6.2	27.9 ± 5.4	26.4 ± 2.8
Basal metabolic rate (kcal)	1655.6 ± 211.0	1665.3 ± 225.3	1654.5 ± 225.5	1647.4 ± 175.8	1457.9 ± 134.6
Visceral fat rating	10.4 ± 4.4	11.0 ± 4.7	10.0 ± 4.5	10.8 ± 4.4	7.7 ± 2.4

^a^ Age (years), anthropometric characteristics and body composition are shown as mean and standard deviation; ^b^ Low education–ISCED levels 0–2; Medium education—ISCED levels 3–4; High education—ISCED levels 5–8; ^c^ Physical activity level (beneficiaries: 1–5 scale; caregivers: IPAQ; 3–low physical activity, 2—medium physical activity; 1—high physical activity); shown as mode (frequency of mode).

**Table 2:** Energy and macronutrient intake and proportion of participants within recommendations, in total and according to the level of care.

	Daily Intake *					*n* (%) ≥ Recommendations					Dietary Reference Values
	Beneficiaries (*n* = 42 **)	Short-Term Care (*n* = 8)	Intensive Care (*n* = 23)	Comprehensive Care (*n* = 11)	Caregivers (*n* = 18 **)	Beneficiaries (*n* = 42)	Short-Term Care (*n* = 8)	Intensive Care (*n* = 23)	Comprehensive Care (*n* = 11)	Caregivers (*n* = 18)	
Energy ^a^, kcal/day	♀ 1801.4 ± 349.9 ♂ 2115.4 ± 386.9	♀ 1595.3 ± 193.2 ♂ 1644.8 ± 243.9	♀ 1844.0 ± 341.6 ♂ 2216.1 ± 270.7	♀ 1866.4 ± 449.9 ♂ 2236.7 ± 445.8	♀ 1673.4 ± 263.7	♀ 6 (23.1) ♂ 4 (25.0)	♀ 0 (0) ♂ 0 (0)	♀ 4 (26.7) ♂ 2 (25.0)	♀ 2 (33.3) ♂ 2 (33.3)	♀ 2 (11.1)	age 40–49: ♀ 2055 kcal/day; ♂ 2553 kcal/day age 50–59: ♀ 2037 kcal/day; ♂ 2519 kcal/day
Protein, g/day	70.9 ± 15.6	56.0 ± 7.5	74.0 ± 15.5	75.2 ± 14.2	62.3 ± 16.8	32 (76.2)	2 (25.0)	21 (91.3)	9 (81.8)	17 (94.4)	0.66 g/kg bw per day
Total fat ^b^, g/day (% energy)	89.9 ± 21.3 (42.1 ± 4.4)	74.5 ± 12.3 (41.6 ± 4.5)	90.8 ± 18.2 (41.5 ± 4.0)	99.1 ± 27.1 (43 ± 4.9)	72.1 ± 18.5 (38.5 ± 6.5)	2 (4.8)	0 (0)	2 (8.7)	0 (0)	0 (0)	20–35% energy
Saturated fat, g/day (% of fat) (% energy)	32.3 ± 10.6 (35.4 ± 5.3) (14.9 ± 2.9)	23.77 ± 7.6 (31.5 ± 6.5) (13.1 ± 3.29)	32.3 ± 8.4 (35.2 ± 4.8) (14.6 ± 2.6)	38.8 ± 12.8 (38.8 ± 3.3) (16.9 ± 2.2)	26.2 ± 9.6 (35.9 ± 5.4) (13.9 ± 3.8)	-	-	-	-	-	ALAP ^c^
					
						2 (4.8)	1 (12.5)	1 (4.3)	0 (0)	3 (16.7)	<10% energy
Carbohydrates ^b^, g/day (% energy)	213.9 ± 50.9 (44.5 ± 4.9)	183.2 ± 26.5 (45.4 ± 3.7)	221.6 ± 53.3 (44.8 ± 5.5)	220.2 ± 53.8 (43.25 ± 4.7)	198.9 ± 35.2 (47.8 ± 6.8)	19 (45.2)	2 (25.0)	12 (52.2)	5 (45.5)	11 (61.1)	45–60% energy
Fiber ^d^	11.7 ± 4.4	9.5 ± 3.1	12.2 ± 4.2	12.7 ± 37.5	13.1 ± 3.1	0 (0)	0 (0)	0 (0)	0 (0)	0 (0)	25 g/day

* Daily intake is shown as mean and standard deviation. ** Dietary record missing (2 beneficiaries; 1 caregiver). ^a^ Dietary reference values for energy are given as average requirements (ARs) for age and gender. Presented ARs are for mean age and gender. Other requirements according to age and gender: age 18–29: ♀ 2147 kcal/day; age 30–39: ♀ 2072 kcal/day; ♂ 2588 kcal/day; age 60–69: ♀ 1861 kcal/day; ♂ 2305 kcal/day. ARs for energy are provided for moderately active adults (PAL = 1.6). ^b^ The reference intake range (RI) is used for energy-yielding macronutrients, except proteins (g/kg bw per day). It is expressed as the proportion (%) of energy derived from that macronutrient. ^c^ ALAP—as low as possible (https://efsa.onlinelibrary.wiley.com/doi/epdf/10.2903/j.efsa.2010.1461 accessed on 21 June 2023). ^d^ Adequate intake (AI)—the average nutrient level, based on observations or experiments, that is assumed to be adequate for the population’s needs.

**Table 3:** Micronutrient intake and proportion of participants within recommendations, according to the level of care.

	Daily Intake ** (% Daily Recommendations)					Dietary Reference Values * (DRV)
	Beneficiaries (*n* = 42)	Short-Term Care (*n* = 8)	Intensive Care (*n* = 23)	Comprehensive Care (*n* = 11)	Caregivers (*n* = 18)	
Vitamins						
Vitamin A, µg/day	490.8 ± 246.4 (86.1 ± 43.2)	355.1 ± 226.1 (62.3 ± 39.7)	522.6 ± 239.1 (91.7 ± 42.0)	523.1 ± 262.9 (91.77 ± 46.1)	582.0 ± 342.5 (102.1 ± 60.1)	570 µg RE/day
Vitamin E, mg/day	♂ 8.7 ± 3.2 (67.0 ± 24.3) ♀ 10.1 ± 4.8 (92.1 ± 43.3)	♂ 5.7 ± 1.3 (43.7 ± 10.2) ♀ 10.6 ± 7.4 (96.4 ± 67.4)	♂ 10.2 ± 3.0 (78.8 ± 23.1) ♀ 11.3 ± 4.2 (102.9 ± 38.4)	♂ 8.1 ± 2.9 (62.1 ± 22.7) ♀ 6.8 ± 1.2 (61.5 ± 11.2)	10.5 ± 3.1 (95.2 ± 28.0)	♂ 13 mg/day ♀ 11 mg/day
Vitamin D, µg/day	3.5 ± 2.9 (23.5 ± 19.6)	4.1 ± 3.6 (27.6 ± 23.7)	3.5 ± 3.2 (23.3 ± 21.2)	3.2 ± 1.9 (20.99 ± 12.9)	6.3 ± 9.1 (42.1 ± 60.3)	15 μg/day
Vitamin K, µg/day	68.5 ± 84.7 (97.8 ± 120.97)	95.2 ± 89.6 (136.0 ± 128.0)	56.4 ± 69.7 (80.6 ± 99.6)	74.3 ± 110.2 (106.2 ± 157.5)	112.5 ± 92.5 (160.7 ± 132.1)	70 μg/day
Thiamine (B_1_) ^b^, mg/day	♂ 1.7 ± 0.5 (222.4 ± 67.6) ♀ 1.6 ± 0.5 (260.2 ± 82.6)	♂ 1.1 ± 0.3 (142.4 ± 39.4) ♀ 1.4 ± 0.4 (223.0 ± 71.7)	♂ 1.9 ± 0.5 (242.3 ± 64.1) ♀ 1.7 ± 0.6 (268.5 ± 92.5)	♂1.8 ± 0.4 (238,5 ± 57.0) ♀ 1.7 ± 0.4 (270.5 ± 65.8)	♀ 1.5 ± 0.5 (239.3 ± 87.3)	♂ 0.77 mg/day ♀ 0.62 mg/day
Riboflavin (B_2_), mg/day	1.6 ± 0.6 (121.7 ± 45.1)	1.0 ± 0.3 (72.9 ± 23.0)	1.7 ± 0.5 (131.9 ± 40.4)	1.8 ± 0.6 (135.8 ± 45.1)	1.30 ± 0.59 (99.7 ± 45.7)	1.3 mg/day
Niacin (B_3_) ^a^, mg/day	♂ 18.2 ± 4.8 (130.3 ± 34.6) ♀ 17.8 ± 6.3 (157.5 ± 55.8)	♂ 13.4 ± 2.6 (95.5 ± 18.7) ♀ 15.4 ± 6.4 (136.3 ± 56.4)	♂ 21.3 ± 4.1 (152.3 ± 29.6) ♀ 19.7 ± 6.7 (174.0 ± 59.6)	♂ 16.2 ± 3.6 (116.0 ± 25.7) ♀ 15.1 ± 3.8 (133.9 ± 33.7)	15.2 ± 4.7 (134.3 ± 41.1)	♂ 14.0 mg/day ♀ 11.3 mg/day
Pantothenic acid (B_5_), mg/day	3.0 ± 1.3 (59.4 ± 26.4)	2.5 ± 1.2 (50.2 ± 23.1)	3.4 ± 1.5 (67.5 ± 29.9)	2.5 ± 0.7 (49.4 ± 13.6)	2.8 ± 1.4 (56.2 ± 27.8)	5 mg/day
Pyridoxine (B_6_), mg/day	♂ 1.8 ± 0.5 (120.5 ± 32.2) ♀ 1.7 ± 0.5 (133.6 ± 39.6)	♂ 1.1 ± 0.3 (71.3 ± 17.1) ♀ 1.3 ± 0.3 (101.0 ± 21.6)	♂ 2.0 ± 0.4 (135.8 ± 24.2) ♀ 1.9 ± 0.6 (148.7 ± 42.8)	♂ 1.9 ± 0.3 (125.5 ± 19.9) ♀ 1.6 ± 0.3 (123.0 ± 21.1)	1.8 ± 0.7 (136.4 ± 54.5)	♂ 1.5 mg/day ♀ 1.3 mg/day
Biotin, μg/day	17.4 ± 7.4 (43.6 ± 18.4)	12.5 ± 4.2 (31.3 ± 10.4)	19.8 ± 7.9 (49.5 ± 19.8)	16.1 ± 6.1 (40.2 ± 15.2)	45.1 ± 108.6 (112.8 ± 271.4)	40 μg/day
Folate (B_9_), μg/day	222.2 ± 68.9 (88.9 ± 27.6)	197.0 ± 57.0 (78.8 ± 22.8)	225.6 ± 65.4 (90.2 ± 26.2)	233.4 ± 84.4 (93.3 ± 33.8)	195.8 ± 68.4 (78.3 ± 27.4)	250 μg/day
Cobalamin (B_12_), μg/day	5.7 ± 5.0 (143.6 ± 124.9)	4.6 ± 4.3 (116.1 ± 106.3)	6.3 ± 5.6 (157.0 ± 139.3)	5.43 ± 4.4 (135.7 ± 110.9)	2.25 ± 1.8 (56.3 ± 45.7)	4 μg/day
Vitamin C, mg/day	♂ 58.9 ± 34.6 (65.4 ± 38.4) ♀ 73.4 ± 61.9 (91.7 ± 77.4)	♂ 33.7 ± 11.8 (37.4 ± 13.1) ♀ 38.6 ± 24.6 (48.3 ± 30.8)	♂ 70.0 ± 43.0 (77.8 ± 47.7) ♀ 95.7 ± 72.1 (119.7 ± 90.2)	♂ 56.2 ± 21.1 (62.5 ± 23.4) ♀ 46.4 ± 22.6 (58.0 ± 28.3)	148.9 ± 181.4 (186.1 ± 226.8)	♂ 90 mg/day ♀ 80 mg/day
Minerals						
Sodium, g/day	3.7 ± 0.9 (184.3 ± 47.2)	2.8 ± 0.6 (140.3 ± 30.6)	3.7 ± 0.8 (184.9 ± 38.5)	4.3 ± 1.0 (215.3 ± 51.2)	3.0 ± 1.1 (151.0 ± 54.7)	2 g/day
Potassium, mg/day	2389.7 ± 603.4 (68.3 ± 17.2)	1712.6 ± 385.3 (48.9 ± 11.0)	2535.6 ± 539.8 (72.4 ± 15.4)	2577.1 ± 541.7 (73.6 ± 15.5)	2379.9 ± 399.2 (68.0 ± 11.4)	3500 mg/day
Calcium, mg/day	1015.9 ± 312.5 (135.5 ± 41.7)	742.9 ± 164.9 (99.1 ± 22.0)	1019.3 ± 302.2 (135.9 ± 40.3)	1207.4 ± 283.6 (161.0 ± 37.8)	844.4 ± 315.7 (112.6 ± 42.1)	750 mg/day
Magnesium, mg/day	♂ 271.8 ± 68.5 (77.7 ± 19.6) ♀ 217.2 ± 51.2 (72.4 ± 17.1)	♂ 168.9 ± 29.5 (48.3 ± 8.4) ♀ 192.0 ± 32.0 (64.0 ± 10.7)	♂ 306.2 ± 53.7 (87.5 ± 15.4) ♀ 224.8 ± 48.8 (74.9 ± 16.3)	♂ 278.7 ± 41.4 (79.6 ± 11.8) ♀ 219.1 ± 69.1 (73.0 ± 23.0)	253.9 ± 125.4 (84.6 ± 41.8)	♂ 350 mg/day ♀ 300 mg/day
Phosphorus, mg/day	1179.1 ± 280.4 (214.4 ± 51.0)	904.7 ± 123.5 (164.5 ± 22.5)	1184.3 ± 258.9 (215.3 ± 47.1)	1367.8 ± 254.4 (248.7 ± 46.3)	1040.1 ± 290.7 (189.1 ± 52.9)	550 mg/day
Iron, mg/day	♂ 11.5 ± 3.5 (192.4 ± 57.6) ♀ 10.1 ± 3.2 (143.9 ± 46.4)	♂ 7.2 ± 0.0 (119.3 ± 0.6) ♀ 7.9 ± 0.7 (113.0 ± 9.5)	♂ 11.8 ± 2.3 (195.8 ± 39.0) ♀ 10.7 ± 3.3 (152.9 ± 47.3)	♂ 13.9 ± 3.7 (230.8 ± 62.4) ♀ 10.3 ± 4.0 (147.0 ± 56.6)	8.3 ± 1.5 (118.4 ± 21.8)	♀ 7 mg/day ♂ 6 mg/day
Copper, mg/day	♂ 1.2 ± 0.3 (73.0 ± 17.3) ♀ 1.0 ± 0.4 (79.5 ± 30.4)	♂ 0.9 ± 0.1 (72.3 ± 9.6) ♀ 0.9 ± 0.2 (56.8 ± 9.9)	♂ 1.2 ± 0.2 (71.7 ± 11.9) ♀ 1.1 ± 0.5 (83.1 ± 35.2)	♂ 1.4 ± 0.3 (84.8 ± 21.1) ♀ 1.0 ± 0.4 (76.4 ± 31.1)	0.9 ± 0.2 (68.5 ± 14.7)	♂ 1.6 mg/day ♀ 1.3 mg/day
Zinc, mg/day	♂ 8.6 ± 2.7 (92.5 ± 28.7) ♀ 7.5 ± 3.0 (99.2 ± 39.5)	♂ 5.1 ± 0.2 (54.5 ± 1.9) ♀ 5.5 ± 0.6 (72.2 ± 7.9)	♂ 9.4 ± 2.4 (100.9 ± 25.5) ♀ 8.1 ± 3.6 (106.9 ± 46.7)	♂ 9.5 ± 2.3 (101.9 ± 24.9) ♀ 7.8 ± 2.0 (102.3 ± 26.7)	7.1 ± 2.6 (93.9 ± 34.6)	♂ 9.3 mg/day ♀ 7.6 mg/day
Chloride, g/day	3.9 ± 1.3 (126.5 ± 41.4)	3.0 ± 0.7 (96.6 ± 23.7)	4.0 ± 1.3 (128.0 ± 42.4)	4.5 ± 1.2 (145.0 ± 40.0)	3.2 ± 1.3 (103.4 ± 43.2)	3.1 g/day
Manganese, mg/day	2.1 ± 0.7 (68.6 ± 21.6)	1.8 ± 0.4 (59.8 ± 12.5)	2.1 ± 0.6 (69.5 ± 21.4)	2.2 ± 0.8 (73.3 ± 26.7)	1.9 ± 0.7 (62.6 ± 21.8)	3 mg/day
Selenium, μg/day	26.8 ± 11.6 (38.3 ± 16.6)	27.5 ± 5.7 (39.3 ± 8.2)	28.4 ± 14.4 (40.6 ± 20.6)	23.1 ± 7.2 (33.0 ± 12.3)	26.5 ± 16.1 (37.9 ± 23.0)	70 μg/day
Iodine, μg/day	47.3 ± 17.4 (31.6 ± 11.6)	34.1 ± 5.3 (22.7 ± 3.5)	50.9 ± 19.5 (33.9 ± 13.0)	49.6 ± 14.2 (33.0 ± 9.5)	42.9 ± 25.1 (28.6 ± 16.7)	150 μg/day

* Dietary Reference Values (DRVs) for the EU. Values calculated for adult (45 years), male (♂) and female (♀). ** Daily intake shown as mean and standard deviation. ^a^ Average Requirement (AR) for adults of 1.3 mg NE/MJ (5.5 mg NE/1000 kcal). Calculated for daily energy requirements according to mean age and gender (♀ 2055 kcal/day; ♂ 2553 kcal/day). ^b^ Average requirement (AR) of 0.072 mg/MJ (0.3 mg/1000 kcal). Calculated for daily energy requirements according to mean age and gender (♀ 2055 kcal/day; ♂ 2553 kcal/day).

## Data Availability

All collected data was entered into an electronic database, and every participant was assigned with a unique code to ensure GDPR compliance. The database is available at Josip Juraj Strossmayer University of Osijek, Faculty of Food Technology Osijek, Department of Food and Nutrition Research.
